# Segmentation of macular neovascularization and leakage in fluorescein angiography images in neovascular age-related macular degeneration using deep learning

**DOI:** 10.1038/s41433-022-02156-6

**Published:** 2022-07-01

**Authors:** David Holomcik, Philipp Seeböck, Bianca S. Gerendas, Georgios Mylonas, Bilal Haj Najeeb, Ursula Schmidt-Erfurth, Gabor Deak

**Affiliations:** Department of Ophthalmology and Optometry, Medical University of Vienna, Vienna, Austria

**Keywords:** Retinal diseases, Macular degeneration, Medical imaging

## Abstract

**Background/objectives:**

We aim to develop an objective fully automated Artificial intelligence (AI) algorithm for MNV lesion size and leakage area segmentation on fluorescein angiography (FA) in patients with neovascular age-related macular degeneration (nAMD).

**Subjects/methods:**

Two FA image datasets collected form large prospective multicentre trials consisting of 4710 images from 513 patients and 4558 images from 514 patients were used to develop and evaluate a deep learning-based algorithm to detect CNV lesion size and leakage area automatically. Manual segmentation of was performed by certified FA graders of the Vienna Reading Center. Precision, Recall and F1 score between AI predictions and manual annotations were computed. In addition, two masked retina experts conducted a clinical-applicability evaluation, comparing the quality of AI based and manual segmentations.

**Results:**

For CNV lesion size and leakage area segmentation, we obtained F1 scores of 0.73 and 0.65, respectively. Expert review resulted in a slight preference for the automated segmentations in both datasets. The quality of automated segmentations was slightly more often judged as good compared to manual annotations.

**Conclusions:**

CNV lesion size and leakage area can be segmented by our automated model at human-level performance, its output being well-accepted during clinical applicability testing. The results provide proof-of-concept that an automated deep learning approach can improve efficacy of objective biomarker analysis in FA images and will be well-suited for clinical application.

## Introduction

Age-related macular degeneration (AMD) is the leading cause for vision loss affecting the population over 50 in the developed world [1].

Retina specialists rely heavily on retinal imaging in the diagnosis, monitoring and therapy of patients with neovascular AMD (nAMD). Although optical coherence tomography (OCT) and in recent years OCT Angiography (OCTA) became the most common diagnostic modalities for diagnosis and monitoring, fluorescein angiography (FA) is still considered as the gold standard of solidifying the diagnosis of nAMD [2]. The most important biomarker of macular neovascularization (MNV) activity seen in FA is leakage area, which is characterized by increasing hyperfluorescence originating from an active MNV lesion seen in late phase images [3].

FA biomarkers such as presence, size and location of CNV lesions as well as its lesion type and activity are very useful in the clinical diagnostics and research [4, 5].

FA evaluation is especially critical in the context of clinical studies for determining study eligibility of patients, tracking disease progression or analyzing the efficacy of treatment. This requires manual segmentation, which is time-consuming, subjective as it suffers from high inter- and intra-grader variability and has to be performed by retina experts [6]. Particularly in large multicentre studies, these drawbacks are very significant, as the process of manual annotation does not scale well with big data, and needs to be harmonized among multiple retina experts within as well as across different reading centres involved in each study. Therefore, developing an automated and objective way to detect and segment CNV lesion size and leakage area is desirable. This would not only allow analyzing FA images in real-time, but also offers the possibility of conducting even large-scale analyses fast and objective to reduce variability of manual readings. Deep-learning based convolutional neural networks (CNN) have already been successfully applied to a multitude of medical imaging tasks outside of ophthalmology such as vessel [7] or brain tissue segmentation [8], and this technique has also shown impressive performance in multiple retinal imaging tasks [9].

In this study, we developed deep learning-based models for fully automated segmentation of CNV lesion size and leakage area. The proposed method additionally provides an uncertainty estimation of the prediction, being important for adopting into clinical routines and eventual subsequent revision by humans [10].

## Materials/subjects and methods

### Dataset

This study was conducted in adherence to the tenets of the Declaration of Helsinki, and ethics approval was obtained by the Ethics Committee of the Medical University of Vienna Submission Nr 1246/2016. The study is a retrospective data analysis and the ethics committee decided that informed patient consent is not necessary.

In this study, two datasets from large prospective multicentre trials for evaluation of anti-vascular endothelial growth factor drugs in patients with nAMD were used, consisting of FA images from the Vienna Reading Center (VRC) image database. The first dataset ‘FA-CNV’ is comprised of 4710 FA images containing CNV lesions from 513 eyes of 513 patients. The second dataset ‘FA-Leakage’ consists of 4558 FA images from 514 eyes of 514 patients.

Manual ground truth image segmentation was performed by certified and trained FA graders of the VRC in a standardized manner to reach best possible human objectiveness. CNV lesion size was annotated in ‘FA-CNV’ and leakage area was annotated in ‘FA-Leakage’ dataset for each individual image. The images to be segmented were selected manually from the best images of the FA image series between 45–90 s for CNV lesions and 5–10 min for leakage area.

All FA images in the datasets have been acquired using different conventional FA cameras certified by the VRC following a standardized image acquisition protocol and fulfilling VRC’s minimal resolution criteria. In particular, the FA images were acquired using FF540+, Spectralis HRA + OCT, TRC50 & TRC50IX, Topcon 2000 and Megaplus 1,4 l from manufacturers Zeiss, Heidelberg, Topcon, Megaplus and Optovue.

No images were excluded due to bad quality. Instead, all images that had been manually annotated were also used for training the automated segmentation model. The used datasets are thus characterized by a large variability in terms of image quality, pixel size, field-of-view, contrast, viewing angle, imaging artefacts or motion blur.

### Technical setup: Network architecture

We propose an AI-based approach using a convolutional neural network (CNN) architecture requiring labelled data [11].

**Fig. 1:**
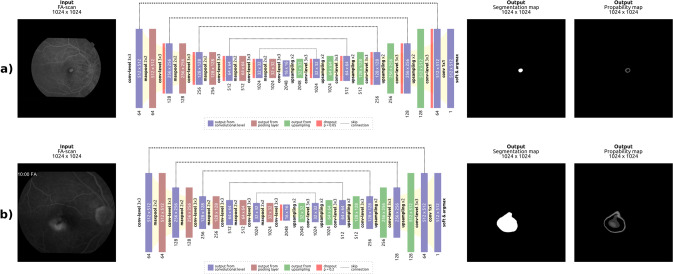
Overview of the network. Overview of the proposed deep learning model for (**a**) CNV lesion size and (**b**) leakage area segmentation. The input image is shown on the left-hand side, while the model output is illustrated on the right hand side, consisting of a binary segmentation and an uncertainty map. The model provides an uncertainty estimation of the prediction for each pixel.

In general, the model follows a U-Net [12] based architecture, with some adaptations. It consists of a contracting (encoder) and expanding (decoder) part, as shown in Fig. 1. Skip connections are used between and within each level, as they have shown to be beneficial for the overall performance of the network [13].

In order to facilitate annotations during clinical application, the proposed model also provides an uncertainty map. For each pixel the model both outputs a prediction and an uncertainty estimation of this prediction [14] (Fig. 1). In this context, uncertainty estimates quantify the confidence of the model regarding its prediction, indicating when and where we can trust the prediction of the model.

### Experimental setup and statistical analysis

Both datasets were randomly split on a patient-distinct basis into 70% training, 10% validation, and 20% test set. While the training set was used for learning the optimal parameters of the model, the validation set was utilized to tune the hyperparameter settings such as the learning rate, number of model parameters or the dropout rate. The final generalization performance of the algorithm was evaluated in the test set. The split resulted in 3343/507/860 training/validation/test FA images in the ‘FA-CNV’ dataset and 3210/493/860 in the ‘FA-Leakage’ dataset.

We assessed the networks segmentation performance by comparing the model predictions with the manual annotations. F1 score, Precision and Recall were used for quantitative evaluation, which are well-known and widely used metrics for comparing segmentation results.

Precision indicates the amount of true positive pixels among all predicted target pixels. Recall is the amount of true positive pixels among all annotated target pixels of the ground truth. In other words, recall is a measure for how good the model is in detecting pixels of the target class, while precision indicates how relevant the detected target pixels are. The F1 score is the harmonic mean of precision and recall used to balance both [15].

We also evaluated the impact of image quality on the segmentation performance of the model. For this we used an internal automated image quality assessment algorithm for FA, providing a score in the range [0,1] for multiple image quality categories (‘overall-quality’, ‘noise’, ‘focus’, ‘contrast’), with 0 indicating bad and 1 good quality. We then computed the Spearman correlation coefficient between these image quality scores and the F1 score of the AI segmentation models on both datasets, across all images in the test set. A description of the automated quality assessment algorithm is provided in the Supplemental information.

### Evaluation of clinical applicability

To evaluate the clinical applicability of the deep-learning model, an extensive evaluation was performed by two trained retina experts of the VRC (GM, BH). For each of the FA images of the test set, both masked experts judged the quality of the manual as well as the automated segmentations as ‘good segmentation’ vs. ‘bad segmentation’. In addition, both experts had to decide which segmentation they preferred for each image. Both experts were masked for this evaluation, meaning that they did not know which image showed the manual annotation and which showed the automated segmentation result coming from the deep learning model.

## Results

### Quantitative and qualitative results

Table 1 shows an overview of the results of the quantitative evaluations including the average F1 score, Precision and Recall for both datasets. The F1 score for the FA-Leakage dataset (0.73) is slightly higher than the F1 score for the FA-CNV dataset (0.65), as are precision and recall. A quantitative comparison of the presented AI algorithm with a baseline is provided in the Supplemental information.

**Table 1:** Depiction of the F1 score, Precision and Recall on ‘FA-CNV and ‘FA-Leakage’ test sets for the final CNV lesion size and leakage area segmentation model.

Dataset	Average F1 score ± standard deviation	Average precision ± standard deviation	Average recall ± standard deviation
FA-CNV	0.65 ± 0.26	0.75 ± 0.29	0.72 ± 0.28
FA-Leakage	0.73 ± 0.21	0.80 ± 0.23	0.78 ± 0.25

On average, the automatic segmentation together with its uncertainty estimation by the AI model required 90 milliseconds of computation time for a single FA image, using a ‘GeForce GTX 1080 Ti’ GPU. The whole segmentation pipeline including loading, pre-processing, segmentation, uncertainty estimation, post-processing and saving of the image took around 3 s, while this pipeline was not optimized for speed.

**Fig. 2:**
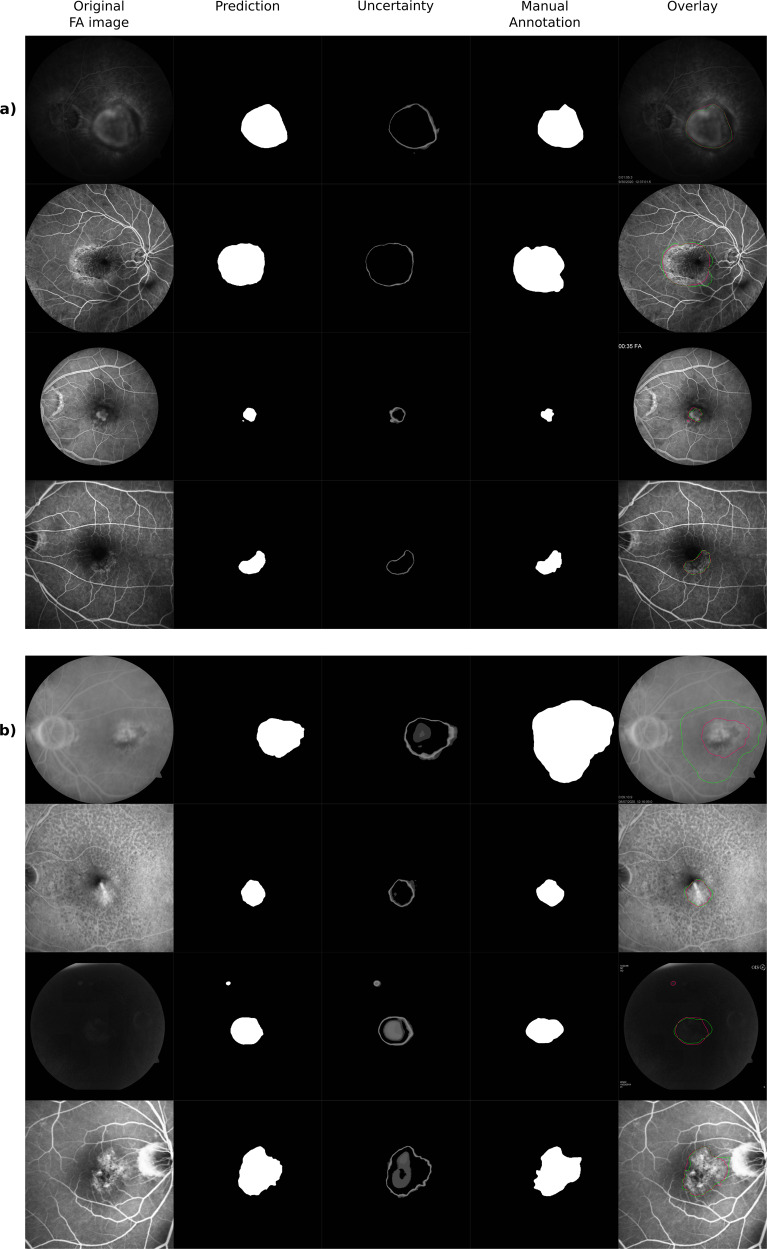
Qualitative results. Qualitative results of (**a**) CNV lesion size and (**b**) Leakage area segmentation. The original FA input image is shown on the left-hand side, followed by the binary automated segmentation map, the uncertainty map, the manual segmentation of the trained experts and the original image with the outline of both manual and automated segmentations overlaid (green: manual annotations, pink: AI prediction). This figure shows a set of representative cases to provide a realistic view on the characteristics of data and results.

Qualitative segmentation results with uncertainty estimates are presented both for the FA-CNV and FA-Leakage dataset in Fig. 2. Typically, the highest uncertainty is present at the edges of segmentations, and in areas of heterogeneous appearance in the centre of the leakage area. Figure 2 depicts a representative set of cases to allow insights into the characteristics of the dataset and results, including the heterogeneity of images (e.g., low contrast in third row of Fig. 2b, differences in field-of-view between third and fourth row in Fig. 2a), improved segmentation of the AI model in comparison with manual annotations (first row of Fig. 2b) or false positive predictions of the algorithm (third row of Fig. 2b).

We observed that the performance of the proposed deep learning models is stable even when facing a wide variety of different input image appearances. This is supported by the Spearman correlation coefficients between the image quality scores and the F1 scores being close to zero: 0.005/0.04/0.02/0.01 in ‘FA-CNV’ and 0.04/0.06/0.07/0.02 in ‘FA-Leakage’ for overall-quality/noise/focus/contrast, respectively.

### Evaluation of clinical applicability

**Fig. 3:**
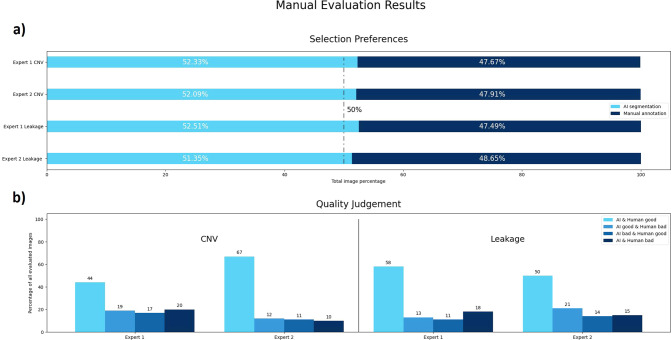
Results of the manual evaluation, performed by two retina experts. **a** Bar graphs illustrate the results for the first judgement, asking which of the shown segmentations is preferred (automated vs. manual in a masked fashion for the grader). **b** Detailed results of the second evaluation, asking if shown segmentations (automated or manual annotation) are of good or bad quality. Available choices were ‘AI & human good’, ‘AI good & human bad’, ‘AI bad & human good’ or ‘AI & human bad’.

The deep learning based automated segmentations of the proposed approach were preferred more often than their human counterparts for both datasets (Fig. 3a).

Both automated predictions and manual human annotations were judged as being of good quality in the majority of cases (Fig. 3b). Compared to manual human annotations, automated predictions were judged more often as good quality, indicating that the proposed deep learning models achieve at least human-level performance in both the ‘FA-CNV’ and the ‘FA-Leakage’ datasets.

## Discussion

In order to streamline the segmentation of CNV lesions and their FA leakage areas, a new deep learning-based approach using uncertainty estimation was proposed. The models produce segmentations in a fast, precise, and upscale-able way, achieving human-level performance in our clinical applicability evaluation.

Manual annotations suffer from a wide variety of problems, such as the time it takes to produce high-quality annotations or the subjectivity leading to high inter-, or intra-reader variability. In contrast, automated models are objective and able to segment in real time, offering the potential for big data analysis and for producing high-quality segmentations even in small or understaffed clinics.

In recent years OCT and OCT angiography (OCTA) more or less replaced FA in routine clinical applications, as these examinations are non-invasive, and faster than FA. Although for the clinical diagnosis of a treatment-naïve CNV, OCT and OCTA are sufficient in most cases, in a fair number of cases FA is still necessary, as only this examination depicts the dynamics of dye leakage over time. Furthermore, although wide-field OCTA systems are being developed, ultra-wide-field FA systems are readily available, and thus are far superior in detecting changes outside of the macular area [16].

Together with signs of exudation in OCT, FA parameters such as CNV lesion size and area of FA leakage are still highly important biomarkers in monitoring disease activity and recurrence during anti-VEGF therapy, and as such are key part of any clinical trial investigating the efficacy of new drugs. Currently such analyses are performed manually by trained graders. Although the graders in our study are certified from a highly standardized renown centre, the VRC, perform numerous alignment trainings, and their gradings follow standardized protocols, inter- and intra-grader reproducibility of such readings is still only moderate to fair [17, 18]. Artificial intelligence (AI) can further improve the reproducibility of the grading of these biomarkers. In future research, combining multiple imaging modalities resulting in high-quality information about the CNV lesion could be achieved. For example, by localizing potential lesions using FA in 2D first, and secondly getting more detailed information by using 3D OCTA. In this context, the proposed model could be also used in future studies to identify potential still unknown „patterns of leakage“ in OCTA by automatically segmenting leakage areas in FA and mapping the corresponding area to OCTA, in datasets where both FA and OCTA are available. Mapping the automatic segmentation in FA to OCTA would allow training an automatic leakage segmentation model for OCTA, learning “leakage patterns” in OCTA without involving manual labels. This may help to replace invasive FA with non-invasive OCTA, that allows faster acquisition, and carries no risk of the potentially adverse reactions [16].

For CNV segmentation, deep learning-based segmentation has been proposed in prior publications for OCTA scans only [19]. For FA images, parametric modelling was used to segment CNV lesions, the algorithm however requiring FA images of each phase, leading to failure in case of non-providence [5]. In contrast, in our work we propose a fully automated segmentation model that operates on individual FA images, providing an output segmentation map using a single FA image as input. On the one hand, this overcomes the drawback of needing multiple images of several phases as input for the segmentation model. On the other hand, this also means that capturing the leakage dynamic can only be done indirectly by segmenting multiple subsequent images.

In the clinical applicability evaluation our models reached human-level performance, meaning that more automated segmentations than manual human segmentations were preferred by the retina experts. With an overall selection rate for ‘FA-Leakage’ of 52.2% and ‘FA-CNV’ of 51.9%, the predictions of the proposed deep learning-based models can be considered as high-quality segmentations on-par with human annotators. The results show that the automated predictions even slightly outperform the manual segmentations in our clinical applicability evaluation. This is a remarkable outcome, especially as the used datasets were particularly challenging due to the heterogeneous composition featuring images of different sharpness, contrast, resolution, field-of-view, etc.

The proposed models allow producing uncertainty estimations simultaneously with its predictions, providing valuable visualizations for subsequent processing or usage. The visualized uncertainty maps in Fig. 2 align with our expectations, being high in border regions and areas of low contrast or heterogeneous appearance. In general, uncertainty maps support the procedure of human revision and offer insights when and where the prediction should be re-evaluated. We believe that this may be a cornerstone of clinical application, helping to open the black box of a convolutional network a bit.

The results did not show a correlation between image quality and segmentation accuracy, meaning that the performance of the AI models was robust with respect to image quality. This demonstrates the potential of the algorithms, as this correlation has been shown to be present with humans [20]. In the application scenario where a subsequent check of the automated segmentation result by a human is intended, an image quality detection algorithm could be utilized as a pre-processing step to ensure a certain quality of the overall pipeline [21, 22].

While both segmentation models show stable and accurate results in general, one limitation is that the models tend to over-segment target areas in complex cases such as multiple pathologies appearing within the same image. However, we also observed that these cases show high uncertainty at the same time and could therefore be identified in clinical practice. Examples are provided in the Supplemental information. Compared to undersegmentation, oversegmentation is less severe, as no lesion would be missed (e.g., in a screening setting). Nevertheless, this limitation with respect to oversegmentation could be addressed in future work by using more training data featuring multiple pathologies, or by using a specific loss function during training that penalizes oversegmentation of the model in a stronger way. One key aspect of FA evaluation in patients with CNV is the determination of dynamic leakage compared with static staining. Our AI algorithm cannot determine this directly, and can only give indirect indication of a dynamic process by providing an accurately measured growth in the area covered by hyperfluorescence when being applied to multiple subsequent images (indicating leakage). Further development is needed to be able to differentiate between these two angiographic features automatically.

In conclusion, we have presented a deep learning-based approach for segmenting CNV lesion sizes and leakage area in individual FA images. The additional capability of the method to provide uncertainty estimations of the automatically created predictions constitutes a key concept for clinical applicability besides real-time performance and scalability. Moreover, the results showing human-level performance of the proposed models in both tasks demonstrate the potential of the method for clinical practice.

## Summary

### What was known before

- The reproducibility of manual grading in FA is moderate to low.
- Due to low intra- and inter-grader reproducibility, the usefulness of biomarkers seen in FA cannot be fully deployed, particularly in the comparison of novel therapies.
- Manual grading is also time-consuming and costly, hindering analyses on a large-scale basis.

### What this study adds

- A new AI segmentation model that provides a fully automated, fast and precise segmentation of CNV lesions and leakage area in FA.
- The model also provides an uncertainty estimation to quantify the confidence of the model regarding its prediction, allowing better interpretability.
- The deep learning algorithm could have largest positive impact on the reproducibility of FA grading, particularly in the setting of randomized multicentre trials.

## Supplementary information

Supplementary Information

## Data Availability

The datasets generated during and/or analyzed during the current study are not publicly available due to privacy restrictions but are available from the corresponding author on reasonable request.
