# Supplementary material for: Segmentation of macular neovascularization and leakage in fluorescein angiography images in neovascular age-related macular degeneration using deep learning
Source: Eye (Lond). 2022 Jul 1;37(7):1439–44. doi: 10.1038/s41433-022-02156-6 (PMC10169785; doi:10.1038/s41433-022-02156-6)
Supplement: Supplementary file 1 — Supplementary Information [file 41433_2022_2156_MOESM1_ESM.docx]

**Supplementary Information for**

**Segmentation of Macular Neovascularization and Leakage in Fluorescein Angiography Images in Neovascular Age-Related Macular Degeneration using Deep Learning**

David Holomcik, BSc; Philipp Seeböck, PhD; Bianca S. Gerendas, MD, PhD; Georgios Mylonas, MD, PhD; Bilal Haj Najeeb MD; Ursula Schmidt-Erfurth, MD, Gabor Deak, MD

**Table of content:**

- **Supplemental text:** Methods Details, Additional Results
- **Supplemental tables:** eTable 1
- **Supplemental figures:** eFigure 1, eFigure 2
- **Supplemental References**

**Methods Details**

**Pre-Processing of FA Scans** Before being fed to the neural network, all images were resized to 1024x1024. To keep the aspect ratio during resizing, the larger side of the image was resized to 1024 pixels while the smaller one was adjusted accordingly. In order to reach a size of (1024x1024), black pixels were added on the side of the image.

Since the majority of images within the datasets feature a black circle on the edges of the image, this black circle was added on top of each image in order to equalize all images. During training, a (512x512) image was randomly cropped out of the resized image, which was then fed to the network for training. During test time no cropping was applied.

**Dataset Split** Both datasets were randomly split into 70% training, 10% validation and 20% test set on a patient distinct basis. The ‘FA-Leakage’ dataset consisted of 3210/493/855 images while the ‘FA-CNV’ dataset consisted of 3343/507/860 images.

**Segmentation Models** For both datasets two separate models were trained. Both models consisted of 6 levels, adapting feature maps of 512x512x54 down to 32x32x2024 in the bottleneck layer. Every level consisted of two subsequent convolutional blocks, each composed of a convolutional layer (kernel size of 3, stride of 1 and padding of 1) followed by batch normalizations and ReLU functions (eFigure 1). Additional skip connections were used at each level, as visualized in eFigure 1. In the encoder max-pooling (kernel size of 2, stride of 2 and padding of 0) was used for downsampling, while an upsample operation (nearest neighbor upsampling with a scale factor of 2) was used in the decoder.

For the ‘FA-Leakage’ model, a single dropout layer (p = 0.2) was present in the bottleneck. For the ‘FA-CNV’ model, dropout layers (p = 0.05) were present after every level, except in the bottleneck and final output. Both models are visualized in Figure 1 of the main manuscript.

To create the final segmentation prediction, the mean of 25 FA segmentations (with turned on dropout during test time) of the same FA image was computed. To obtain the corresponding uncertainty estimation, the standard deviation over these 25 predictions was calculated for each pixel.

**Training Details** Due to hardware constraints, training was done using a batch size of 4. Both models were trained for 50 epochs (for ‘FA-Leakage’ 160500 iterations, for ‘FA-CNV’ 167150 iterations) using the Adam optimizer with standard parameters, a learning rate of 10^-4^ and CrossEntropyLoss as loss function.

The only data augmentation strategy applied during the training stage of the model was random cropping with (512 x 512) pixels.

**Evaluation Details** During evaluation, no augmentation methods were utilized. Monte Carlo dropout sampling was only applied during evaluation, not during training.

**Image Quality Assessment Algorithm** The internal FA quality assessment algorithm is an AI based approach, predicting the probability of good/bad quality of FA images for multiple categories (‘contrast’, ‘focus’, ‘noise’, ‘overall quality’). In particular, the model provides a quality score between 0 and 1 for each specific category. The architecture of this convolutional neural network (CNN) follows a ResNet-18 architecture with four residual blocks (1) and extra dropout layers added after each block. The model was trained and evaluated using an internal dataset with FA images and manual labels of the four image quality categories provided by the Vienna Reading Center (VRC) from large prospective multicentre trials.

**Additional Results**

**Baseline segmentation based on threshold** In order to investigate if the AI algorithm indeed learned the semantic context of the retina and the disease in the FA images, we conducted a comparison with a simple threshold-based segmentation baseline. In particular, for an input image, all pixels with a grayscale value higher than the threshold were segmented as target of interest, and all pixels with a value below as background. We optimized the threshold utilizing the training set: As the FA images were rescaled to the range [0,1], we varied the thresholds from 0.01 to 0.99 (0.01 steps) and computed the average F1 score on the training set for each value. The threshold with the highest F1 score on the training set was then used as the final value and applied to the test set (threshold for CNV: 0.12, threshold for Leakage: 0.25). The quantitative results in comparison with the developed AI algorithm are depicted in eTable 1, with the baseline reaching F1 scores of 0.01 and 0.16 for CNV and Leakage segmentation, respectively. The results show that the AI models clearly outperform this simple baseline by a large margin. This indicates that the networks do not only rely on a simple thresholding or gray level based segmentation, but instead learn the semantic context in the FA images.

|  | Average F1 - CNV | Average F1 - Leakage |
| --- | --- | --- |
| Baseline (Threshold-based) | 0.01 | 0.16 |
| AI Algorithm | 0.65 | 0.73 |

eTable 1: Quantitative results of both the AI algorithm and the threshold-based baseline segmentation.

**Supplemental Figures**


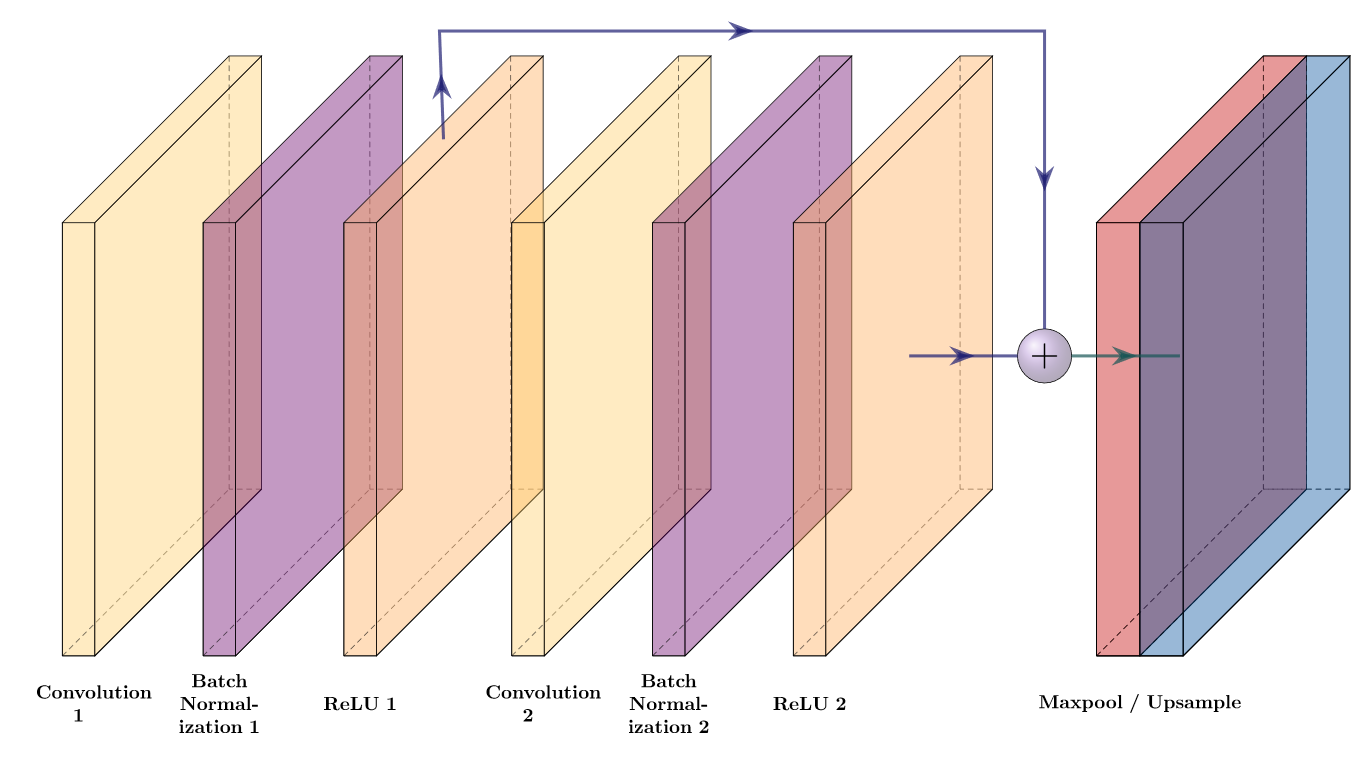


eFigure 1: Illustration of the convolutional block architecture, utilized on individual levels of the network. It consists of two subsequent sets of convolutional operations, batch normalization and ReLU. Skip connections are present between both ReLUs and combined by addition before being either fed into the maxpool or upsampling layer.


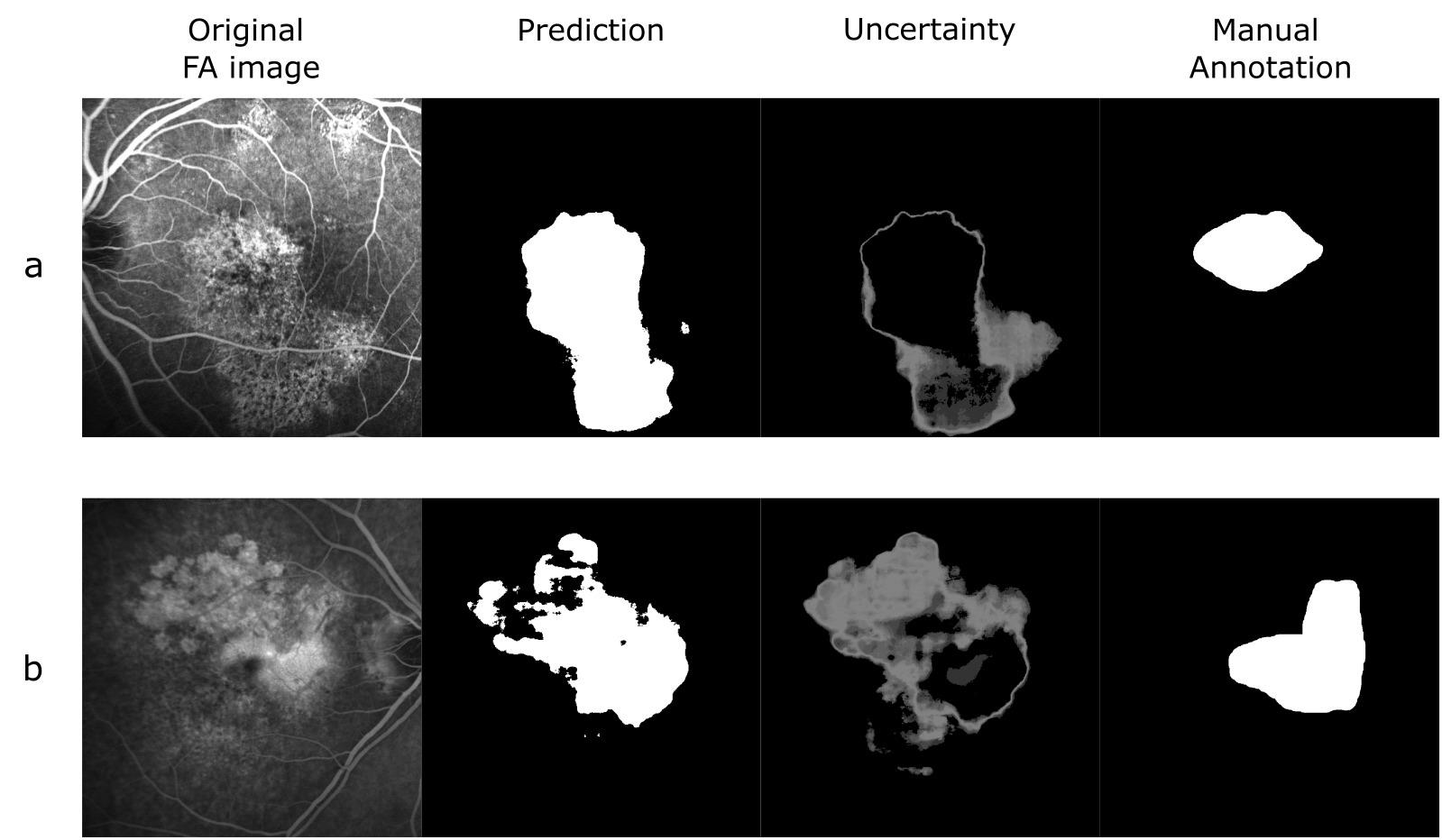


eFigure 2: Two cases for severe oversegmentation of the deep learning model. a) Irregular hyperfluorescence due to RPE damage was oversegmented by the algorithm as part of the CNV lesion. At the same time, the model showed high uncertainty in this area. b) Example of leakage segmentation with patches of window-defect due to retinal pigment epithelium (RPE) atrophy that was oversegmented as leakage. Again, high uncertainty was observed in this area.

**Supplemental References**x

| 1. | He K, Zhang X, Ren S, Sun J. Deep residual learning for image recognition. In Proceedings of the IEEE conference on computer vision and pattern recognition; 2016. p. 770–778. |
| --- | --- |

x
